# From Anatomical Staging to Precision Prognostication: Evolution of Gallbladder Cancer Staging Across AJCC Editions and the 2025 UICC TNM Update

**DOI:** 10.3390/jpm16080396

**Published:** 2026-07-24

**Authors:** Tianyi Shen, Nicholas Kai Yi Tan, Wen Xuan Chew, Matthias Yi Quan Liau, Vor Luvira, Vinay K. Kapoor, Orestis Ioannidis, Vishal G. Shelat

**Affiliations:** 1 Lee Kong Chian School of Medicine, Nanyang Technological University, Singapore 308232, Singapore; tracyshen930@gmail.com (T.S.); nic.tan.ky@gmail.com (N.K.Y.T.);; 2 Department of Surgery, Faculty of Medicine, Khon Kaen University, Khon Kaen 40002, Thailand; 3 Department of Surgical Gastroenterology, Mahatma Gandhi Medical College and Hospital (MGMCH), Mahatma Gandhi University of Medical Sciences and Technology (MGUMST), Jaipur 302 022, India; 4 4th Department of Surgery, Medical School, General Hospital “George Papanikolaou”, Aristotle University of Thessaloniki, 57010 Thessaloniki, Greece; iorestis@auth.gr; 5 Department of General Surgery, Tan Tock Seng Hospital, Singapore 308433, Singapore

**Keywords:** AJCC, gallbladder cancer, molecular markers, personalized management, prognostication, TNM staging

## Abstract

Gallbladder cancer (GBC) is the most common biliary tract malignancy and is among the most lethal gastrointestinal cancers. Since its inclusion in cancer staging manuals, the GBC Tumour–Node–Metastasis (TNM) classification has undergone repeated revisions intended to improve anatomical precision and prognostic discrimination. This narrative review traces the evolution of GBC staging across AJCC editions and the 2025 UICC TNM update and critically examines whether these refinements have translated into clinically meaningful risk stratification. In the 2025 UICC TNM 9 grouping, Stage IA corresponds to T1aN0M0 and Stage IB to T1bN0M0, while T2aN0M0 and T2bN0M0 remain Stage IIA and IIB, respectively. Structured literature searches were performed to identify historical staging documents, population-based analyses, validation studies, and prognostic reports relevant to stage migration, survival discrimination, and emerging staging modifiers. Successive changes, including T-category refinement and the transition from nodal location to nodal burden, have improved anatomical resolution. However, available validation studies suggest that gains in overall prognostic performance remain modest, with reported concordance indices often remaining near 0.66. Contemporary evidence increasingly shows that tumour location, adequacy of lymphadenectomy, number of positive lymph nodes, lymph node ratio, log odds of positive lymph nodes, obstructive jaundice, residual disease status, and selected molecular alterations may capture inter-patient heterogeneity beyond anatomy alone. Future staging refinement should therefore preserve TNM as a common anatomical language while developing validated parallel modifiers that support more individualized prognostication and treatment selection.

## 1. Introduction

Gallbladder cancer (GBC) is the most common biliary tract malignancy and remains a highly lethal gastrointestinal cancer [1]. GBC has an aggressive tumour biology with early metastatic spread and dismal survival prognosis [2]. Nevin et al. were the first to advocate a depth of invasion-based staging system based on a retrospective review of 66 patients and distinguished lymph node metastases from distant metastases [3]. The American Joint Committee on Cancer (AJCC) staging manual provides the modern contemporary Tumour, Node, Metastasis (TNM) staging framework for GBC and evolves with emerging evidence [4]. Historical surgical series have shown that patients with liver hilum or hepatoduodenal ligament involvement often have surgically advanced disease and poorer candidacy for curative resection [5]. Distant metastatic sites for GBCs typically include the liver, peritoneum, lung and pleura [4]. Common risk factors of GBC include female sex, obesity, a personal or family history of cholelithiasis, abnormal pancreatiobiliary ductal junction, and chronic gallbladder inflammation [2].

Despite being first described by Maxmillan de Stol in 1777 [6], GBC is still difficult to manage and prognosticate. The AJCC Cancer Staging Manual provides the most widely used contemporary Tumour–Node–Metastasis framework for GBC, with the 8th edition remaining the current AJCC gallbladder cancer staging reference unless replaced by a disease-site-specific AJCC Version 9 protocol [4]. In parallel, the UICC has released the 2025 TNM ninth edition. This review therefore distinguishes AJCC gallbladder staging evolution from the 2025 UICC TNM update. These revisions reflect an evolving understanding of GBC’s heterogeneous biology and metastatic behaviour. Despite advances in imaging and surgical techniques, prognostic assessment in GBC continues to rely heavily on anatomical parameters that incompletely reflect tumour biology and distinct carcinogenic pathways. ICPN, a WHO-recognized preinvasive gallbladder neoplasm, illustrates that GBC can arise through pathways beyond flat dysplasia–carcinoma progression [7]. Furthermore, an improved understanding of gene mutations, such as K-RAS, TP53 and c-ERB-B2, and gene promoter hypermethylation with associated impacts on prognosis has contributed to a better understanding of the disease [8]. Given the cumulative revisions across editions, a consolidated understanding of what has changed and why may illuminate the broader conceptual trajectory of GBC staging. Beyond documenting chronological changes, this review critically examines whether successive staging refinements have translated into meaningful prognostic and clinical utility and identifies evidence-based directions for future refinement.

The objectives of this review are twofold: first, to examine how emerging clinicopathologic evidence has shaped successive AJCC TNM revisions for GBC; and second, to evaluate whether these revisions have meaningfully improved prognostic stratification at the individual-patient level. GBC is a useful test case for personalized medicine because patients with similar anatomical TNM stage may have markedly different tumour biology, resectability, recurrence risk, and therapeutic opportunities. In this review, we therefore examine AJCC stage evolution not only as a historical sequence of anatomic refinements but also through a personalized-medicine lens: whether emerging non-anatomic modifiers such as tumour location, adequacy of lymphadenectomy, lymph node ratio, log odds of positive lymph nodes, obstructive jaundice, and molecular alterations can improve individual risk stratification and better support treatment selection beyond conventional TNM staging alone.

## 2. Methods

This study is a narrative, historically anchored review of gallbladder cancer staging, informed by structured literature searches. It was not designed as a formal systematic review or meta-analysis. The aim was to describe and critically appraise the evolution of GBC staging across AJCC editions and the 2025 UICC TNM update and to relate these staging changes to contemporary evidence on prognostic discrimination, surgical decision-making, and personalized risk stratification.

A structured literature search was performed in PubMed, Web of Science, Scopus, and Embase from database inception to 8 February 2025. Search terms included combinations of “gallbladder cancer,” “carcinoma of the gallbladder,” “biliary tract neoplasm,” “biliary malignancy,” “AJCC,” “TNM,” “UICC,” “cancer staging,” “stage migration,” “prognosis,” “survival,” “recurrence,” “nodal spread,” “lymph node metastasis,” “lymph node ratio,” “metastases,” “vascular invasion,” and “perineural invasion.” Detailed search strings for each database are provided in Supplement S1.

Records were eligible for consideration if they addressed GBC staging, AJCC or UICC TNM classification, prognostic validation of staging systems, stage migration, lymph node assessment, surgical prognostic factors, or candidate non-anatomical modifiers relevant to future staging. We prioritized staging manuals, population-based studies, registry analyses, multicentre cohorts, validation studies, and clinically relevant prognostic studies. Studies were excluded if they focused exclusively on non-epithelial gallbladder tumours, cancers arising primarily outside the gallbladder, non-English publications without accessible data, reports without survival or staging relevance, or duplicate cohorts where a larger or more complete report was available.

The search identified 1873 records. After duplicate removal, 1171 records remained. Title and abstract screening excluded 935 records because they were not focused on GBC, did not address AJCC/UICC/TNM staging or prognostication, were non-English, or could not be retrieved. Full texts were assessed for 236 records. Of these, 191 were excluded because they were not focused on GBC, did not address staging or prognosis, contained no survival or prognostic data relevant to the review question, represented abstract-only reports, or duplicated previously reported cohorts. Forty-five studies were included in the qualitative synthesis. Reference lists of the included articles were also manually searched to identify additional historical staging documents and landmark prognostic studies. This process did not identify additional eligible records beyond those already captured through the database searches.

Data were extracted narratively with attention to staging edition, study design, data source, sample size, staging variables, survival outcomes, C-index or other discrimination metrics where reported, and relevance to anatomical or non-anatomical prognostic modifiers. Because the included sources consisted mainly of staging manuals, registry analyses, retrospective cohorts, and narrative or validation studies rather than interventional trials, formal risk-of-bias scoring was not applied. Instead, methodological features were appraised qualitatively, including data source, cohort size, completeness of nodal assessment, use of multivariable analysis, and risk of stage migration or treatment selection bias.

**Figure 1:**
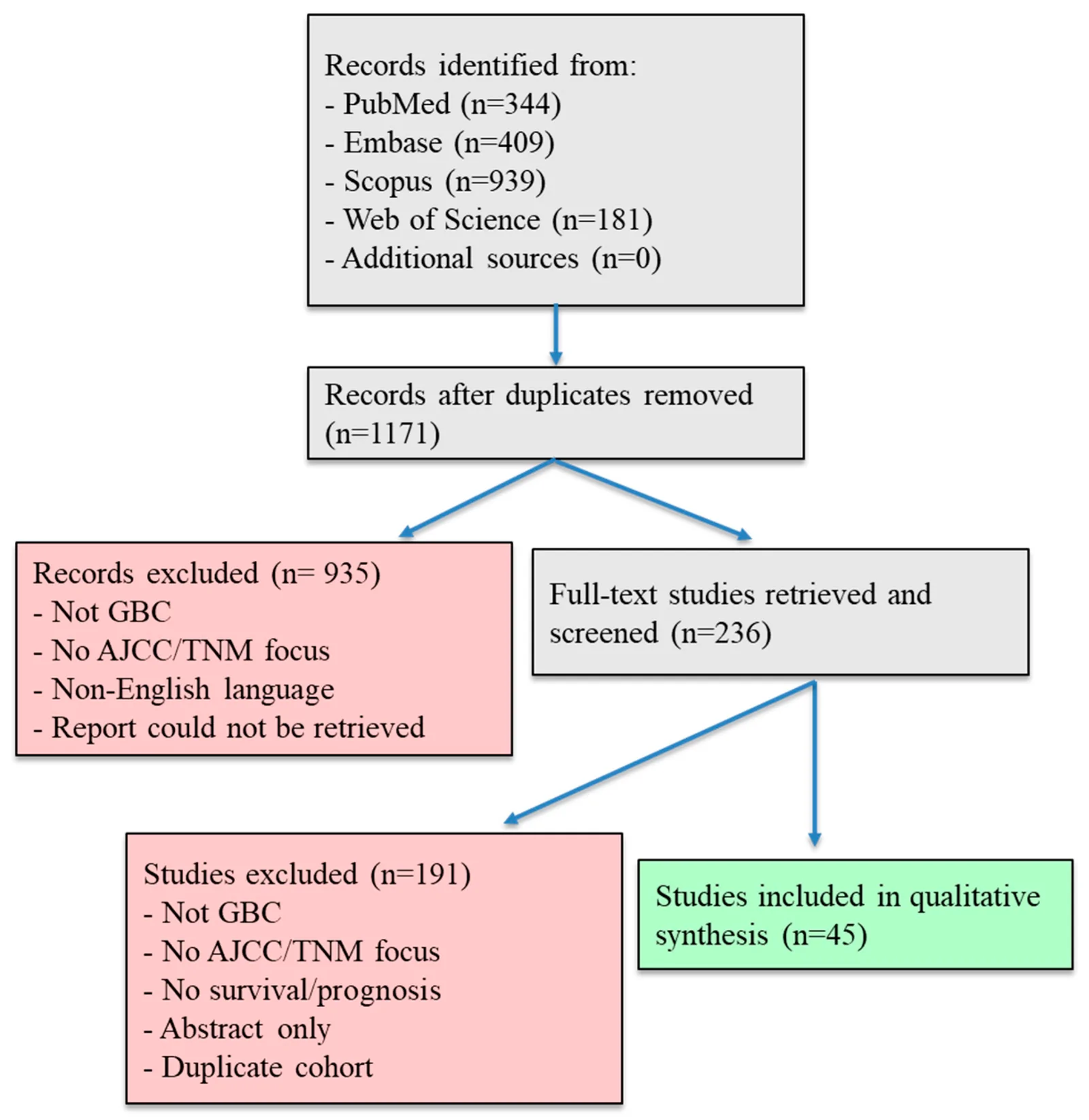
Literature identification and selection flow for this structured narrative review.

**Figure 2:**
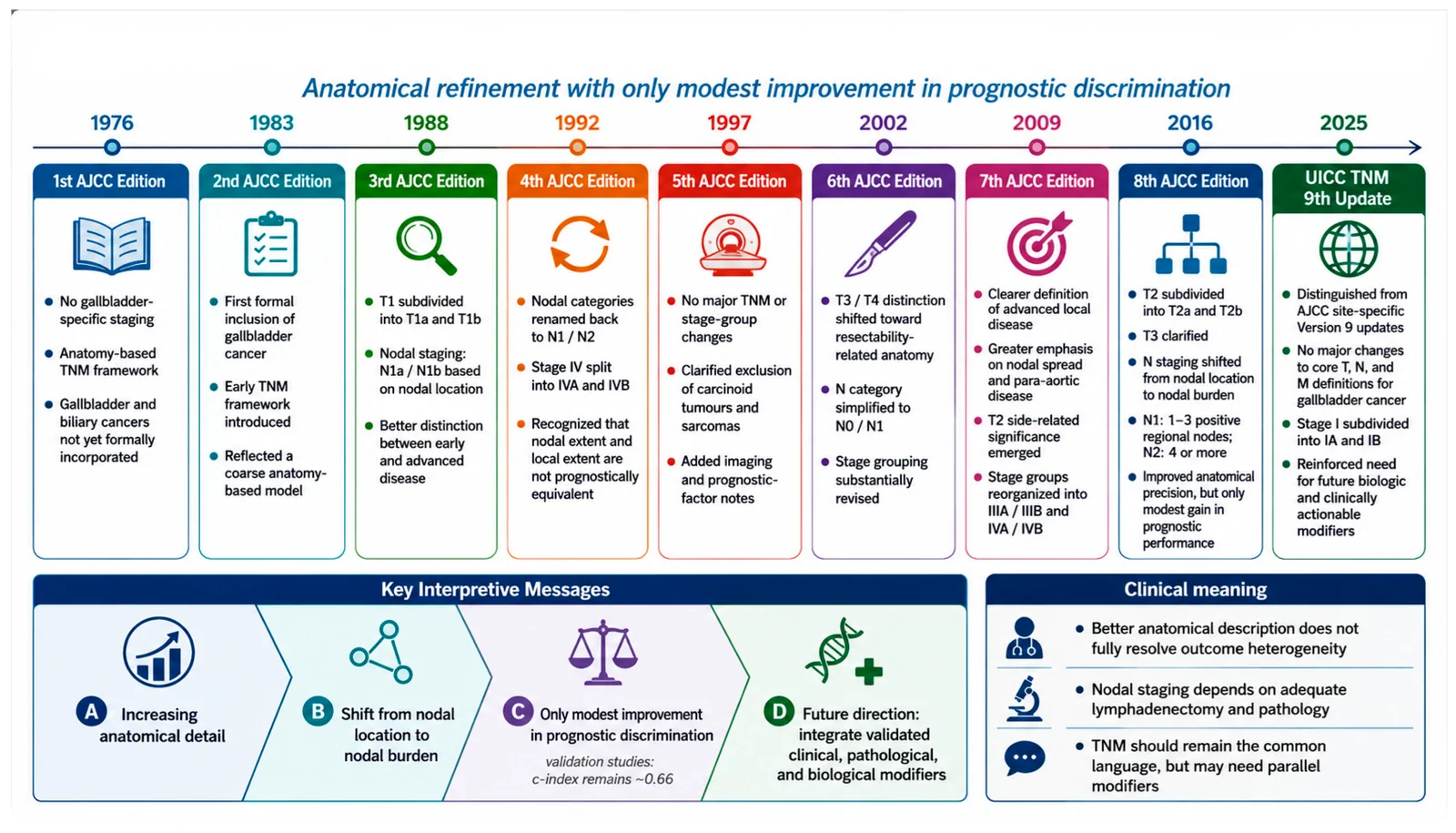
Evolution of gallbladder cancer TNM staging: AJCC editions and the 2025 UICC update.

**Figure 3:**
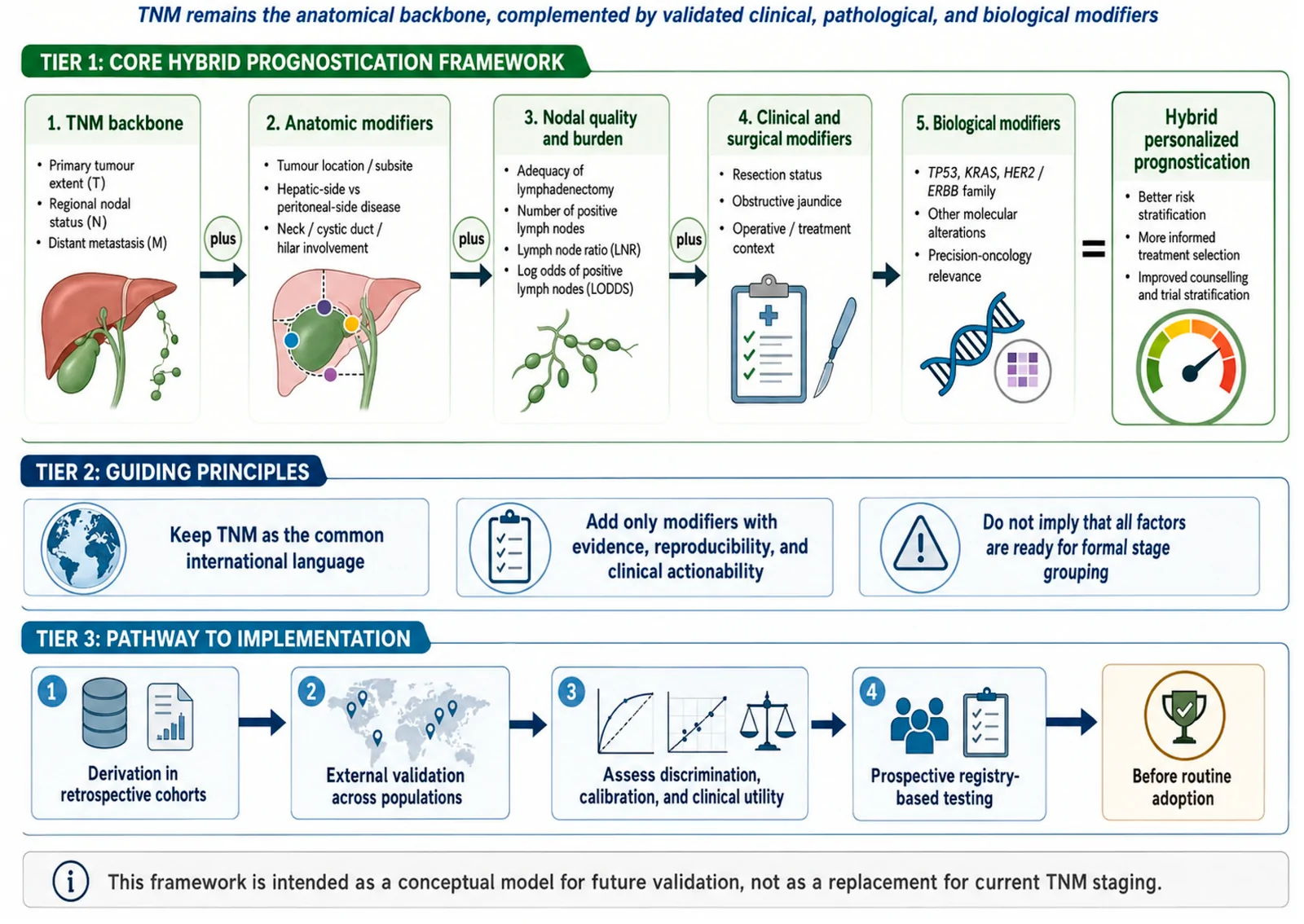
Proposed hybrid personalized prognostication framework for gallbladder cancer.

Figure 1 summarizes the literature identification and selection flow for this structured narrative review. The authors used ChatGPT version 5.5 to generate Figure 2 and Figure 3. All AI-assisted content was reviewed, edited, and verified by the authors, who take full responsibility for the final manuscript.

Studies that focused exclusively on non-epithelial tumours or on primary cancers arising in other organs were excluded. Rare non-adenocarcinoma subtypes of GBC were also excluded from the main synthesis. Examples include gallbladder carcinosarcomas with fewer than 100 known cases globally [9] and primary gallbladder lymphomas, which remain uncommon in clinical practice [10].

### Definitions

Gallbladder Cancer (GBC): The staging manual includes primary gallbladder adenocarcinoma with the tumour epicentre in the gallbladder. GBC can be picked up through incidental findings (such as post-cholecystectomy) and as symptomatic cases.

Overall Survival Concordance Index (OS C-index): This index measures the probability that the model correctly predicts the survival results. If a model predicting OS in cancer patients has a C-index of 0.60, the model correctly orders OS times for 60% of comparable patient pairs. Higher C-index values generally indicate better risk stratification.

Lymph Node Ratio (LNR): The ratio of metastatic (positive) lymph nodes to the total number of resected and examined lymph nodes.

Log Odds of Positive Lymph Nodes (LODDS): It is defined as the log of the ratio between positive and negative nodes (with a correction factor to avoid zero values) [11]. For consistency, “nodal burden” is used as an umbrella term for the extent of nodal disease, while “number of positive lymph nodes,” “lymph node ratio,” and “LODDS” are used only when referring to those specific nodal metrics.

## 3. 1st Edition

The AJCC developed the TNM system as a standardized approach to describe cancer extent and guide prognosis, initially relying solely on anatomical assessment. By the late 1990s, the AJCC began recognizing the importance of non-anatomic prognostic factors such as molecular and genetic markers, and convened a Prognostic Factors Consensus Conference to evaluate their incorporation [12]. The 1st edition included staging for gastrointestinal organs but omitted the liver, gallbladder, and biliary tract, aside from a brief reference to the common bile duct in pancreatic diagrams [13]. This likely reflected the limited GBC research at the time; the Nevin classification subsequently informed its introduction in the 2nd edition [3]. Research progress has remained constrained by the paradox of high GBC prevalence in low-income settings and low prevalence in high-income regions with stronger research infrastructure [14]. Further, marked geographic and socioeconomic heterogeneity in GBC burden, with substantial variation in incidence and mortality across countries and regions, supports the perspective on slow progress in GBC staging systems [15,16].

## 4. 2nd Edition

The 2nd edition of the AJCC staging manual was published in 1983. It marks the first time cancers of the liver and biliary tract, the extrahepatic bile ducts, and the gallbladder were introduced in the manual [17].

The T1 definition referenced invasion into the ‘submucosa,’ although the gallbladder wall lacks a true submucosal layer [18]; this reflected early histopathologic assumptions later corrected in subsequent editions. The histopathological staging frameworks that informed the T-classification, including carcinoma in situ and microinvasive carcinoma, were reported by Bivins BA et al. [19]. Additionally, the schema appears not to account for early T1–T2 tumours with nodal metastasis, implying that any nodal involvement is treated as a feature of advanced (Stage III or higher) GBC. This gap likely contributed to understaging biologically aggressive early tumours, a theme that persisted across later staging revisions and remains relevant to contemporary TNM classification [20].

## 5. 3rd to 5th Editions—Early Evolutions of GBC Staging

The TNM staging for GBC received minor revisions from the 3rd to the 5th editions as discussed by Fong Y et al. [21]. In the 3rd edition, T1 was separated into T1a (invades mucosa) and T1b (invades muscular layer), in line with the gallbladder’s histological anatomy [22]. In the N category, N1 and N2 were changed to N1a and N1b. Nodes in the cystic duct, pericholedochal, and/or hilar lymph nodes (i.e., in the hepatoduodenal ligament) were classified as N1a, and those in the peripancreatic (head only), periduodenal, periportal, celiac, and/or superior mesenteric lymph nodes were classified as N1b.

Past studies suggest that lymphatic spread in GBC first involves the cystic and pericholedochal lymph nodes (LNs) [23,24]. Further spread involves the hepatoduodenal ligament, with downstream nodal involvement towards the posteriosuperior pancreaticoduodenal, retroportal, celiac, and interaorticocaval LNs [25].

While the stage classification for GBC remained stable from the 2nd to the 3rd edition, the stage grouping was altered slightly, with T2 being separated out and indicated as part of Stage II GBC. Stage III GBCs were classified as the moment any nodal involvement was present. Stage IV disease was more explicitly defined to include T4 tumours with any nodal status and without distant metastases or any tumour with distant metastasis (M1) regardless of T or N classification. This represented a shift from the broader criteria in the 2nd edition, where Stage IV encompassed all T3/T4 tumours with nodal involvement and distant metastases, toward a more streamlined and metastasis-driven definition.

In the 4th edition, N1a and N1b were renamed back to N1 and N2, respectively. In the stage grouping, Stage IV was subdivided into Stage IVA, which included T4 N0/N1 M0, and Stage IVB, which included any tumour with N2 or M1. One significant implication of this was that GBC with N2, excluding T4 tumours, would now be considered Stage IVB, up from Stage III previously. The 4th edition also updated the rules of classification with recognition that some patients may not have resected pathology specimens available for staging and advanced the concept of both clinical staging as well as pathologic staging.

An evaluation of the trends in GBC treatment and prognosis in the National Cancer Database concluded that survival was proportionally related to cancer stage, with a 60.4% 5-year survival rate for Stage 0 tumours, 38.9% for Stage I tumours, 14.5% for Stage II tumours, 5.3% for Stage III tumours, and only 1.1% for Stage IV tumours [26]. The frequency of LN metastasis also correlated well with the local extent of tumour, with 48% nodal involvement in T2 tumours, 72% nodal involvement in T3 tumours, and 80% nodal involvement in T4 tumours [26]. In fact, T3 and T4 tumours were found to have greater N2 nodal involvement in comparison to T2 tumours [27,28]. This was the possible reason for upstaging GBC with N2 involvement from Stage III in the 3rd edition to Stage IV in the 4th edition. These data provided the first robust evidence that anatomical extent and nodal status were not interchangeable prognostic determinants.

The 5th edition added three clarifications not present in the 4th edition. Firstly, in the title segment, it stated non-inclusion of carcinoid tumours and sarcoma from the staging system. Secondly, in the clinical staging segment, it added ultrasound or computed tomography imaging as enablers. Finally, it added a concluding paragraph on prognostic factors. The TNM considerations and stage grouping remained unchanged from the previous 4th edition. The limited changes likely reflected the slow evolution of diagnostic, therapeutic, and outcome evidence during that period [29,30].

## 6. 6th to 8th Editions—Refinement of the Prognostic Abilities of the Manual

The 6th edition mentions the summary of changes box at the beginning for ease of readers. The T3 and T4 distinction was now not based on the depth of liver invasion but on the potential for resectability with T3 deemed as potentially resectable and T4 as unresectable. It introduced T4 tumours as having direct invasion into the portal vein, hepatic artery, or multiple extrahepatic organs or structures. This change aimed to better distinguish unresectable cases due to major vascular involvement. Additionally, it differentiated invasion into the liver versus other extrahepatic structures, improving staging precision. Lymph node (N) classification remained simple with N0 and N1 for absent or present regional lymph node spread, respectively. This binary approach facilitated clinical decision-making but did not account for nodal burden or distribution. The stage grouping was changed substantially from the 5th edition, with Stage 1A (T1N0M0), Stage 1B (T2N0M0), Stage IIA (T3N0M0) and Stage IIB (T1-3N1M0). This serves to downstage 5th edition Stage II to revised stage IB, stage III T3N0 to revised stage IIA, and stage III T1-3N1 to stage IIB in the 6th edition. The role of lymphadenectomy and aggressive surgical resection in improved survival outcomes was increasingly reported and validated [31,32]. Furthermore, stage IVA and IVB were unified to a single stage IV, which included M1 disease. However, the result showed no significant improvement in the discrimination power of staging between the 5th and 6th editions. Lastly, the 6th edition also mentioned the role of magnetic resonance imaging in clinical staging, as well as the consideration of repeat surgery in patients operated for incidentally discovered GBC.

The 7th edition proposed four key changes compared to the 6th edition. Firstly, in the T4 stage, clarification is made regarding the involvement of two or more extrahepatic organs or structures (instead of multiple in the 6th edition). Secondly, the coeliac and superior mesenteric artery nodes were categorized as metastatic and not regional. Thirdly, the nodal staging was categorized as N0-N2 with periaortic, pericaval, coeliac, and superior mesenteric artery nodes categorized as N2 with apparent internal inconsistency with metastatic disease. Fourthly, stage grouping was changed with unification of stage 1A/1B and stage IIA/IIB to stage I and stage II, respectively, and the splitting of stage III and stage IV to stage IIIA/IIIB and stage IVA/IVB. It was noted that peritoneal- versus hepatic-side involvement is clinically significant for T2 tumours, and the importance of cystic duct involvement that warrants bile duct resection was also mentioned. This change was based on evidence that hepatic-side tumours have worse survival outcomes than peritoneal-side tumours [33]. Hepatic-side tumours had higher rates of vascular invasion (51 vs. 19%), neural invasion (33 vs. 8%), and nodal metastasis (40 vs. 17%) than tumours on the peritoneal side (*p* < 0.01 for all) [33]. Hepatic-side tumours had worse 3-year (52.1 vs. 73.7%) and 5-year (42.6 vs. 64.7%) survival rates compared to peritoneal-side tumours [33]. Studies have shown significant differences in the 5-year survival rate and LN recurrence rate among different numbers of LNs involved [34,35]. A retrospective study of 60 patients undergoing radical resection with para-aortic lymphadenectomy reported that patients with para-aortic disease showed poor postoperative survival, similar to those with distant metastasis. This led to the conclusion that para-aortic nodal spread behaves more similarly to distant metastasis, and hence, the treatment involved should be different [36]. This reclassification reinforced the distinction between locoregional progression and biologically systemic disease. The prognostic relevance of this expanded nodal stratification was evaluated by Oh et al. in a single-institution cohort of 142 patients, where the AJCC 7th edition demonstrated clear survival separation between N1 and N2 disease, with a median overall survival of 56.1 months for N1 versus 27.6 months for N2 (*p* = 0.006) [37]. Notably, the survival distinction between N0 and N1 became statistically significant only after excluding patients who had not undergone lymphadenectomy, underscoring how inadequate nodal assessment can mask the intended discriminatory effect of nodal reclassification. These findings support the biological rationale for incorporating nodal burden into staging while highlighting its dependence on surgical completeness and pathological evaluation. Lastly, the pathologic staging section of the 7th edition also mentions the resection classification categorization (e.g., R0, R1, etc.) as a stage-independent prognostic factor and encouraged the reporting of this.

In the 8th edition, T3 was clarified to specify invasion into the serosa, liver, or adjacent organs. N classification was refined to consider nodal burden, with N1 defined as 1–3 regional nodes and N2 as more than 3 nodes. The revised N classification was validated by the analysis of large databases, which consistently showed that the number of positive LNs was a more powerful prognostic factor than their anatomical locations [38]. Although validation studies found that the nodal classification offered more granularity, it did not significantly improve prognostic performance over the 7th edition [39]. To compare the prognostic performance of the 7th and 8th editions and assess the impact of the revised nodal criteria, investigators analysed 7743 GBC cases, with 202 patients reclassified under the 8th edition. Relative mortality across editions and within new strata showed no improvement in prognostic accuracy, with similar OS C-indices for the AJCC 8th (0.665) and 7th editions (0.663) [39]. These findings confirmed that even substantial changes to nodal categorization may not overcome intrinsic biological heterogeneity.

## 7. 2025 UICC TNM Ninth Edition Update and Its Relationship to AJCC GBC Staging

The 2025 UICC TNM 9th edition introduced a limited but important refinement to early GBC stage grouping, while AJCC GBC staging remains based on the AJCC 8th edition unless superseded by a disease-site-specific AJCC Version 9 protocol [4,20]. This distinction is important because the terminology “ninth edition” refers to the UICC TNM manual rather than an AJCC gallbladder-specific revision. In UICC TNM 9, Stage IA corresponds to T1aN0M0 and Stage IB to T1bN0M0, whereas T2aN0M0 and T2bN0M0 are classified as Stage IIA and Stage IIB, respectively [20]. Thus, the 2025 UICC update separates T1a from T1b disease within early node-negative GBC. Cai et al. showed that T category is the dominant predictor of survival independent of N/M: the median survival was ~8 months for T3 and ~2.7 months for T4 [40]. Although early tumours were a minority in their cohort, lower-T lesions were often incidental and achieved excellent surgical outcomes.

Figure 2 summarizes the evolution of gallbladder cancer TNM staging across AJCC editions and the 2025 UICC TNM update. Overall, GBC staging has progressively refined anatomical descriptors but has not substantially improved prognostic discrimination. T-category changes, including T1 sub-stratification, T2a/b separation, and clarification of T3/T4 definitions, were driven by an improved understanding of anatomic spread, whereas N-category shifts reflect evolving evidence on nodal burden. However, validation studies show limited C-index improvement, highlighting that anatomical staging alone cannot capture underlying tumour biology.

Although Figure 2 summarizes the chronological evolution of AJCC/UICC GBC staging, the key question is whether these anatomical refinements improved prognostic discrimination. Table 1 summarizes representative studies that evaluated survival separation, stage migration, nodal stratification, or model discrimination across AJCC editions and related modified staging approaches.

Taken together, these studies suggest that GBC staging has become progressively more anatomically precise, but available validation data show only modest and sometimes inconsistent improvement in individualized prognostic discrimination. The historical evolution summarized above shows a consistent pattern: each staging revision improved anatomical description, but no single revision fully resolved the mismatch between anatomical extent and individual outcome. The following discussion therefore moves from chronology to interpretation, focusing on why prognostic discrimination remains limited and how future staging may incorporate clinically actionable modifiers without losing the simplicity and reproducibility of TNM.

## 8. Discussion

The evolution of AJCC staging for GBC reflects a broader tension in oncologic classification: the need to preserve a simple, globally reproducible anatomical framework while also capturing the biologic heterogeneity that determines outcomes in individual patients. Across nine editions, AJCC revisions have progressively refined descriptors of local tumour extent and nodal disease, yet these changes have yielded only modest improvements in global prognostic discrimination. This suggests that, in GBC, anatomy alone is an incomplete surrogate for clinically meaningful risk. Together, these shifts highlight both the progress made and the intrinsic limitations of an anatomy-dominant staging system. These shifts paralleled evidence that selected tumours invading the liver could still benefit from radical resection [38]. The late Dr. Blake Cady’s oft-cited observation that “Biology is King” is especially apt in GBC, where aggressive tumour behaviour may outpace both diagnostic resolution and surgical capability.

AJCC/UICC TNM staging and the Japanese Society of Hepato-Biliary-Pancreatic Surgery (JSHBPS) classification serve related but distinct purposes. AJCC/UICC TNM provides a globally reproducible anatomical language for cancer stage grouping, registry reporting, and broad prognostic communication. In contrast, the Japanese classification gives greater emphasis to detailed surgico-pathological description, standardized specimen handling, and topographic mapping of the gallbladder. It records tumour location across the fundus, body, neck, hepatic side, peritoneal side, and circumferential involvement and provides detailed guidance for specimen preparation and assessment [41]. This added granularity is particularly relevant for GBC because hepatic-side disease, neck involvement, cystic duct involvement, and hilar extension may have different prognostic and surgical implications despite similar AJCC/UICC T categories. The Japanese system should therefore be viewed as complementary rather than competing: it can improve pathological reproducibility and anatomical mapping, while AJCC/UICC TNM preserves the common international staging language. Radical resection remains the only potentially curative option for selected locally advanced GBC patients [42]. However, evidence continues to show that escalation to vascular or multi-organ resection rarely improves survival [43,44]. This underscores the limited ability of an anatomy-only staging system to guide complex surgical decision-making. Robotic approaches may facilitate lymphadenectomy, though their long-term oncologic impact remains unclear [45,46].

Although T2a/T2b stratification shows modest differences, distinctions such as IIIA vs. IIIB or IVA vs. IVB remain inconsistent [47]. In contrast, staging systems incorporating positive lymph node ratio (pLNR), tumour grade, or molecular features have shown significantly enhanced prognostic performance [48]. These findings suggest that although finer subcategorization may support registry auditing and retrospective analysis, it does not necessarily translate into clinically actionable stratification, unless accompanied by additional biologic or functional parameters.

## 9. Future Directions for the GBC AJCC Staging

Future refinement of GBC staging should not simply add more anatomical subdivisions. A practical next step is a hybrid prognostic framework that preserves TNM as the anatomical backbone while reporting validated modifiers in parallel. Such a framework should be simple enough for global use and sufficiently informative for individualized counselling, adjuvant treatment decisions, trial stratification, and registry-based outcome analysis. Candidate modifiers can be grouped into three domains: anatomical modifiers, nodal-quality and nodal-burden modifiers, and biological or clinical-actionability modifiers. These domains should not be weighted equally. Tumour location, lymphadenectomy adequacy, number of positive lymph nodes, and resection status currently have stronger evidence and better reproducibility than most molecular markers. Molecular alterations are important for precision oncology, but at present, they are better viewed as parallel biological descriptors or trial-stratification variables rather than as replacements for TNM categories. Figure 3 illustrates a conceptual hybrid personalized prognostication framework for GBC, integrating anatomic, nodal, clinical, surgical, and biological domains, together with the guiding principles and validation pathway required before routine adoption.

### 9.1. Anatomic Burden

Although TNM is fundamentally anatomy-based, not all anatomical information has equal biological or surgical meaning. Emerging evidence suggests that future refinement should focus less on repeated descriptor proliferation and more on those anatomical features that clearly affect outcome and resectability. Tumour location is one such factor. Hepatic-side tumours, proximal or neck lesions, and cystic duct involvement are associated with more advanced behaviour, greater operative complexity, and poorer survival than similarly staged tumours in less adverse locations [33,49,50]. A location-based clinical classification—stratifying abdominal, liver, hilar, and mixed growth patterns—correlates with distinctly different prognoses, with median OS of ≈48, 21, 16, and 11 months, respectively [41]. Likewise, distinctions between technically resectable and biologically unresectable patterns of local extension may be more meaningful than additional broad T-category expansion. Thus, a personalized staging framework should continue to use TNM as its anatomical base while explicitly recognizing that subsite, direction of spread, and structurally critical involvement may refine prognosis within the same nominal T stage.

### 9.2. Biologic Aggressiveness

Biologic aggressiveness includes both clinical–physiological markers and molecular features that may reflect tumour behaviour not captured by anatomical stage alone. Recent studies have examined the associations between overall survival in GBC and clinical or physiological markers, with some models outperforming traditional TNM staging in prognostic accuracy [51,52]. These data support the broader principle that anatomy alone may not fully capture individual risk.

Molecular markers should be interpreted cautiously and separated into prognostic, predictive or actionable, and investigational categories. From a prognostic perspective, alterations involving TP53, KRAS, HER2/ERBB2, ERBB3, and the broader ErbB pathway have been reported in GBC [53,54,55]. Li et al. identified recurrent non-silent mutations in TP53, KRAS, and ERBB3 and found ErbB pathway alterations in 36.8% of GBC [55]. Importantly, ErbB pathway alterations were associated with worse survival independent of TNM stage, suggesting that molecular biology may explain part of the outcome heterogeneity observed within the same anatomical stage [55].

From a predictive or actionable perspective, HER2/ERBB2 alterations are relevant because they may identify a subgroup of patients for precision-oncology strategies, particularly in advanced or recurrent biliary tract cancer [53,54,55,56,57]. Nevertheless, the evidence base in GBC remains less mature than in more common cancers, and therapeutic actionability depends on alteration type, assay method, tumour context, drug availability, and clinical trial access. Therefore, these markers are not ready to define routine anatomical stage, but they may support parallel biological reporting, treatment selection, and trial stratification.

Other molecular features, including PI3K pathway alterations, microsatellite instability-high status, tumour mutational burden, and rare actionable events, remain investigational in the specific context of GBC staging [53,54,55,56]. These markers may eventually support biological classification, but their current role is better framed as complementary to TNM rather than as a replacement for it. Future staging research should test whether molecular markers improve discrimination, calibration, and clinical decision-making when added to TNM, nodal adequacy, tumour location, resection status, and obstructive jaundice.

### 9.3. Clinical Actionability

Nodal assessment illustrates the difference between anatomical classification and clinically useful prognostication. The shift in the 8th edition from nodal location to nodal burden was an important advance, but large studies show that improvements in global discrimination remain modest [38,39]. This suggests that nodal staging may need to be interpreted alongside other clinically actionable variables rather than in isolation. The 8th edition replaced location-based nodal staging with a quantitative system (N1: 1–3 nodes; N2: ≥4 nodes), supported by a recommendation to examine at least six lymph nodes to reduce understaging [21]. However, real-world nodal yields are often modest, and several studies highlight the limitations of the current N1/N2 thresholds. While ≥4 positive nodes correlates with poorer survival, even a single metastatic node substantially worsens prognosis [34,38,58]. In a Surveillance, Epidemiology, and End Results (SEER) cohort of patients above 65 years, any nodal metastasis increased mortality risk more than twofold, and involvement of more than two nodes further amplified hazard [58]. Additional inconsistencies include counterintuitive survival patterns. In SEER and Fudan University Zhongshan Hospital (FUZH) analyses, patients with N2 disease had significantly better survival after R0 resection than those with M1 disease, despite both being classified as stage IVB, and IIIA disease (T3N0M0) demonstrated worse outcomes than IIIB (T1–2N1M0) [59]. These discrepancies support contextualizing nodal burden by T stage and ensuring adequate lymphadenectomy. Current guidelines recommend a D2 dissection (stations 8, 12a/12b/12c/12p, 13a) with retrieval of at least 4–6 nodes for accurate staging [60]. Adequacy of lymphadenectomy is central here, because insufficient nodal harvest can mask true burden and weaken the apparent performance of the N category [21,37,60]. Beyond simple node counts, metrics such as the lymph node ratio and LODDS offer more individualized estimates of nodal risk, especially when nodal yield is variable [11,61,62,63].

Sentinel lymph node (SLN) approaches have been explored but appear applicable only in select subgroups. In a multicentre Japanese study, the cystic duct node (CDN) reliably predicted downstream nodal status only in peritoneal-side tumours (T2p/T3p), with no skipped metastases and superior DFS [64]. In hepatic-side tumours (T2h/T3h), frequent skip metastasis and higher involvement of porta hepatis and common hepatic artery nodes limited SLN reliability. Thus, systematic lymphadenectomy remains essential for hepatic-side lesions and all T3 tumours. Fluorescence-guided SLN biopsy is technically feasible but requires further validation before clinical adoption [65].

Similarly, residual disease status and surgical obstructive jaundice provide clinically meaningful context that influences treatment planning and prognosis even though they sit outside conventional TNM categories [37,66,67,68]. These features may be especially useful as adjunctive reporting elements or parallel modifiers in future staging systems because they are directly relevant to therapeutic decision-making and postoperative counselling. Surgical obstructive jaundice (SOJ) arising either from metastatic lymph nodes in the hepatoduodenal ligament compressing the common bile duct or from direct invasion of the common hepatic/bile duct remains absent from all editions of the AJCC GBC staging system despite its clear prognostic implications. Earlier series demonstrated its adverse impact: in a prospective cohort of 240 patients, 34% presented with jaundice, yet only 7% underwent curative-intent resection, and 5% achieved negative margins, with a median disease-specific survival of 6 months compared with 16 months in non-jaundiced patients and no 2-year disease-free survivors [66]. More contemporary data, however, suggest that SOJ should not be considered an absolute contraindication to resection. In a single-centre cohort of 59 non-metastatic jaundiced patients, curative-intent surgery yielded a markedly longer median survival than non-curative approaches (20 vs. 6 months; *p* = 0.001), with benefits observed with and without chemotherapy [67]. A prospective series of 65 resected patients similarly reported comparable 5-year overall survival between jaundiced and non-jaundiced groups (27% vs. 31%, *p* = 0.742) despite more extensive operations in the jaundiced cohort, supporting aggressive resection in carefully selected non-metastatic SOJ [68]. The candidate modifiers discussed above are summarized in Table 2, together with their supporting evidence, current clinical use, and proposed status in a future personalized prognostic framework.

A hybrid model should be developed in stages rather than introduced directly into formal stage grouping. First, candidate variables should be tested in large retrospective cohorts with adequate data on T category, nodal yield, positive nodes, margin status, tumour location, obstructive jaundice, treatment, and survival. Second, internally derived models should be externally validated across geographic regions and health systems, especially because GBC burden is high in regions that may be under-represented in existing datasets. Third, model performance should be assessed not only by the C-index but also by calibration, net reclassification improvement, decision-curve analysis, and clinical usability. Finally, prospective registry-based validation should test whether the model improves counselling, adjuvant treatment selection, trial stratification, and outcome prediction beyond TNM alone.

## 10. Limitations of This Study

Several limitations of the available evidence should temper interpretation of staging performance comparisons. Much of the evidence comes from retrospective cohorts, registry datasets, and single-institution surgical series. SEER and NCDB analyses provide large sample sizes but may contain incomplete information on lymph node yield, margin status, operative extent, pathological processing, systemic therapy, recurrence, and disease-specific treatment selection. Surgical and pathological practices also vary across institutions and eras, especially in lymphadenectomy and nodal retrieval, which directly affect N classification, LNR, and LODDS. Stage migration is another important concern: improvements in imaging, pathological sampling, and nodal assessment can change apparent stage distribution without necessarily reflecting true biological change. Comparisons across AJCC/UICC editions should therefore be interpreted as evidence of broad prognostic patterns rather than definitive proof that one staging edition fully captures individual biological risk.

## 11. Conclusions

Despite incremental refinements across AJCC editions and the 2025 UICC TNM update, the staging system for GBC continues to offer only limited individualized prognostic discrimination. Contemporary evidence suggests that tumour location, nodal burden, adequacy of lymphadenectomy, and alternative nodal metrics such as the LNR and LODDS often provide greater prognostic insight than conventional N categories alone. In parallel, clinically important features such as obstructive jaundice, residual disease risk, and emerging molecular alterations including TP53, KRAS, and HER2 further illustrate the gap between anatomical stage and true biological behaviour. The central challenge for future staging refinements is therefore not merely to preserve a universal staging language but to complement it with validated modifiers that are reproducible, clinically actionable, and globally feasible. A hybrid system that retains anatomical simplicity while incorporating validated biologic and clinically actionable modifiers may better support individualized risk stratification, therapeutic planning, and outcome prediction in GBC.

## Figures and Tables

**Table 1:** Summary of evidence on prognostic performance and limitations of GBC staging systems.

Study/Data Source	Staging System or Model Evaluated	Key Quantitative Findings and Interpretation for Staging
Donohue et al., NCDB [26]	Earlier AJCC stage groupings	Five-year survival declined by stage: Stage 0, 60.4%; Stage I, 38.9%; Stage II, 14.5%; Stage III, 5.3%; Stage IV, 1.1%. Nodal involvement increased with T category. Anatomical stage correlates with survival, but broad stage groups still contain substantial biological and clinical heterogeneity.
Fong et al., NCDB [21]	AJCC 5th versus 6th edition	The 6th edition changed several stage groupings, including downstaging selected T2, T3N0, and T1–3N1 categories, but did not produce a major improvement in discriminatory performance. Major anatomical regrouping did not necessarily translate into substantial prognostic gain.
Oh et al., single-institution cohort [37]	AJCC 6th versus 7th edition	The 7th edition separated N1 and N2 disease, with a median OS of 56.1 months for N1 versus 27.6 months for N2. The N0 versus N1 distinction became clearer after excluding patients without lymphadenectomy. The nodal stratification’s prognostic utility depends on adequate lymphadenectomy and pathological assessment.
Sakata et al., resected GBC cohort [38]	Number of positive lymph nodes	Number of positive lymph nodes independently predicted prognosis after resection. Supports the later shift from nodal location toward nodal burden.
Giannis et al., population-based validation cohort [39]	AJCC 7th versus 8th edition	OS concordance indices were similar for AJCC 7th and 8th editions: 0.663 versus 0.665. The AJCC 8th nodal burden approach improved anatomical logic but produced only minimal global discrimination gain.

**Abbreviations:** AJCC, American Joint Committee on Cancer; GBC, gallbladder cancer; NCDB, National Cancer Data Base; OS, overall survival;

**Table 2:** Candidate modifiers beyond TNM for personalized prognostication in gallbladder cancer.

Candidate Modifier	What it Adds Beyond TNM	Supporting Evidence	Current Clinical Use	Comment
Tumour location	Captures heterogeneity within similar T stage	Hepatic-side T2 tumours and proximal/neck or cystic duct involvement have been associated with more aggressive behaviour and poorer outcomes [33,49,50]	Used in operative planning and pathological interpretation; not a formal TNM stage component	Hepatic-side and neck/cystic duct disease may behave more aggressively than similarly staged peritoneal-side disease
Adequacy of lymphadenectomy	Indicates confidence in nodal staging	Nodal staging performance depends on lymphadenectomy and pathological assessment; inadequate nodal yield can weaken N classification [21,37,60]	Relevant to surgical quality, pathological staging, and postoperative risk interpretation	Low nodal yield can produce understaging and reduce reliability of N category
Number of positive lymph nodes	Reflects extent of nodal disease	Positive lymph node count has been shown to independently predict prognosis and informed the shift toward nodal burden in AJCC 8th edition [34,38,39]	Incorporated into AJCC 8th edition N classification	More informative than nodal location alone in several datasets
Lymph node ratio	Adjusts nodal risk to total nodal yield	LNR has been evaluated as an alternative nodal metric when nodal harvest varies [61,62,63]	Not routine TNM staging; may support postoperative prognostication	Useful when total number of retrieved nodes differs substantially across patients
LODDS	Integrates positive and negative nodal information	LODDS-based models have been proposed for postoperative prognostication, especially in advanced or node-assessed disease [11,61,62,63]	Investigational/adjunctive prognostic metric	May remain informative when nodal harvest is limited but requires validation before routine use
Resection status	Reflects completeness of tumour clearance	Residual disease status is a stage-independent prognostic factor and is recommended for reporting [37]	Routinely reported pathologically; used in recurrence risk and adjuvant treatment discussions	Better suited as a parallel prognostic descriptor than as a pure anatomical stage component
Surgical obstructive jaundice	Signals adverse local disease biology and complex surgical selection	Obstructive jaundice has adverse prognostic implications, but selected non-metastatic patients may still benefit from curative-intent surgery [66,67,68]	Used in surgical selection and counselling; not part of TNM staging	Should not be treated as an automatic contraindication to resection in all cases
Molecular alterations	Captures biological aggressiveness and potential therapeutic relevance	TP53, KRAS, HER2/ERBB2, ERBB3, and ErbB pathway alterations have been reported in GBC, with emerging prognostic and therapeutic implications [8,53,54,55,56]	Increasingly relevant in advanced/recurrent disease and trial stratification; not ready for formal stage grouping	Best viewed as a parallel biological descriptor until validated for staging performance
Hybrid clinical–biological models	Combines anatomy with clinical, pathological, and biological data	Clinically based or modified models have shown potential to improve prognostic stratification beyond conventional TNM alone [48,52]	Conceptual/future validation priority	More practical than repeatedly expanding anatomy-only stage groups but requires external validation

**Abbreviations:** AJCC, American Joint Committee on Cancer; GBC, gallbladder cancer; LNR, lymph node ratio; LODDS, log odds of positive lymph nodes; TNM, Tumour–Node–Metastasis.

## Data Availability

No new data were created or analysed in this study. Data sharing is not applicable to this article.

## Supplementary Materials

The following supporting information can be downloaded at: https://www.mdpi.com/article/10.3390/jpm16080396/s1, Supplement S1 : Database Search Strategies for Gallbladder Cancer Staging Review.
