# Supplementary material for: From Anatomical Staging to Precision Prognostication: Evolution of Gallbladder Cancer Staging Across AJCC Editions and the 2025 UICC TNM Update
Source: J Pers Med. 2026 Jul 24;16(8):396. doi: 10.3390/jpm16080396 (PMC13514314; doi:10.3390/jpm16080396)
Supplement: Supplementary file 1 [file jpm-16-00396-s001.zip › jpm-4274583-supplementary.pdf]

Supplement S1: Database Search Strategies for Gallbladder Cancer Staging Review

Search coverage: from database inception to 8 February 2025.

This supplement reports the exact Boolean search strings used across PubMed, Embase (Ovid), Scopus, and Web of Science for reproducibility. Search concepts included: disease terms (gallbladder cancer and biliary tract neoplasms), staging frameworks (AJCC, TNM, UICC, neoplasm staging), and prognostic/outcome terms (survival, recurrence, nodal spread, metastases, lymph node ratio, vascular/perineural invasion). Field tags and controlled vocabulary were tailored to each database. Optional database-specific limits (e.g., humans, original research or review articles, English language, and publication date) were considered but applied cautiously to avoid excluding seminal historical AJCC papers.

PubMed

The PubMed strategy leverages the Medical Subject Headings (MeSH) thesaurus for high-relevance concepts while supplementing with free-text terms in the title/abstract fields (tiab) to ensure comprehensive retrieval of recent literature that may not be indexed with MeSH terms.

("Gallbladder Neoplasms"[Mesh] OR "gallbladder carcinoma"[tiab] OR "carcinoma of gallbladder"[tiab] OR "gall bladder cancer"[tiab]) AND ("Neoplasm Staging"[Mesh] OR "AJCC"[tiab] OR "TNM"[tiab] OR "UICC"[tiab] OR "cancer staging"[tiab] OR "stage migration"[tiab] OR "stage grouping"[tiab]) AND ("Prognosis"[Mesh] OR "Survival"[Mesh] OR survival[tiab] OR "overall survival"[tiab] OR prognostic[tiab])

Embase

The Embase search utilizes the Emtree vocabulary, starting with the exploded term 'exp gallbladder carcinoma/', and combines this with a comprehensive set of free-text terms in the title and abstract fields (.ti,ab.) to capture all relevant staging and classification frameworks within the Embase content.

(exp gallbladder carcinoma/ OR "gallbladder carcinoma".ti,ab. OR "carcinoma of gallbladder".ti,ab. OR "gall bladder cancer".ti,ab.) AND ("AJCC".ti,ab. OR "TNM".ti,ab. OR "UICC".ti,ab. OR "cancer staging".ti,ab. OR "stage migration".ti,ab. OR "stage grouping".ti,ab.)

Scopus

The Scopus search was constructed using the TITLE-ABS-KEY function to query terms across the title, abstract, and keywords simultaneously. This strategy employs a broad set of synonyms for the

disease, staging, and prognostic outcomes to thoroughly search Scopus's extensive abstract and citation database.

(TITLE-ABS-KEY("gallbladder cancer" OR "gall bladder cancer" OR "carcinoma of gallbladder" OR "gallbladder carcinoma" OR "biliary tract neoplasm*" OR "biliary malignan*" OR "biliary tract cancer")) AND (TITLE-ABS-KEY("AJCC" OR "TNM" OR "UICC" OR "staging" OR "cancer staging" OR "tumor staging" OR "stage migration" OR "stage grouping" OR "prognostic classification")) AND (TITLE-ABS-KEY("prognosis" OR "survival" OR "overall survival" OR "disease-free survival" OR "recurrence" OR "outcome*" OR "risk factor*" OR "progression" OR "metastases" OR "nodal spread" OR "lymph node metastasis" OR "lymph node ratio" OR "vascular invasion" OR "perineural invasion"))

Web of Science Core Collection

For Web of Science, the Topic (TS) field was used, which searches titles, abstracts, author keywords, and Keywords Plus. This approach ensures a wide capture of relevant records by encompassing the core concepts of disease, staging systems, and prognostic factors across the multidisciplinary index.

TS=("gallbladder cancer" OR "gall bladder cancer" OR "carcinoma of gallbladder" OR "gallbladder carcinoma" OR "biliary tract neoplasm*" OR "biliary malignan*" OR "biliary tract cancer") AND TS=("AJCC" OR "TNM" OR "UICC" OR "staging" OR "cancer staging" OR "tumor staging" OR "stage migration" OR "stage grouping" OR "prognostic classification") AND TS=("prognosis" OR "survival" OR "overall survival" OR "disease-free survival" OR "recurrence" OR "outcome*" OR "risk factor*" OR "progression" OR "metastases" OR "nodal spread" OR "lymph node metastasis" OR "lymph node ratio" OR "vascular invasion" OR "perineural invasion")
